# Alterations in stride-to-stride fluctuations in patients with chronic obstructive pulmonary disease during a self-paced treadmill 6-minute walk test

**DOI:** 10.1371/journal.pone.0300592

**Published:** 2024-03-15

**Authors:** Wai-Yan Liu, Martijn A. Spruit, Jeannet M. Delbressine, Paul J. Willems, Jennifer M. Yentes, Sjoerd M. Bruijn, Frits M. E. Franssen, Emiel F. M. Wouters, Kenneth Meijer

**Affiliations:** 1 Department of Nutrition and Movement Sciences, NUTRIM School of Nutrition and Translational Research in Metabolism, Maastricht University Medical Centre+, Maastricht, The Netherlands; 2 Department of Research and Development, Ciro, Horn, The Netherlands; 3 School of Nutrition and Translational Research in Metabolism (NUTRIM), Maastricht University Medical Centre+, Maastricht, The Netherlands; 4 Department of Respiratory Medicine, Maastricht University Medical Centre+, Maastricht, The Netherlands; 5 Department of Health & Kinesiology, Texas A&M University, College Station, Texas, United States of America; 6 Department of Human Movement Sciences, Vrije Universiteit Amsterdam, Amsterdam, The Netherlands; 7 Department of Orthopedics, First Affiliated Hospital of Fujian Medical University, Fuzhou, Fujian, P.R. China; 8 Ludwig Boltzmann Institute for Lung Health, Vienna, Austria; Kennedy Krieger Institute/Johns Hopkins University School of Medicine, UNITED STATES

## Abstract

Evaluating variability and stability using measures for nonlinear dynamics may provide additional insight into the structure of the locomotor system, reflecting the neuromuscular system’s organization of gait. This is in particular of interest when this system is affected by a respiratory disease and it’s extrapulmonary manifestations. This study assessed stride-to-stride fluctuations and gait stability in patients with chronic obstructive pulmonary disease (COPD) during a self-paced, treadmill 6-minute walk test (6MWT) and its association with clinical outcomes. In this cross-sectional study, eighty patients with COPD (age 62±7y; forced expiratory volume in first second 56±19%predicted) and 39 healthy older adults (62±7y) were analyzed. Gait parameters including stride-to-stride fluctuations (coefficient of variation (CoV), predictability (sample entropy) and stability (Local Divergence Exponent (LDE)) were calculated over spatiotemporal parameters and center of mass velocity. Independent t-test, Mann-Whitney U test and ANCOVA analyses were conducted. Correlations were calculated between gait parameters, functional mobility using Timed Up and Go Test, and quadriceps muscle strength using dynamometry. Patients walked slower than healthy older adults. After correction for Speed, patients demonstrated increased CoV in stride length (F(1,116) = 5.658, *p* = 0.019), and increased stride length predictability (F(1,116) = 3.959, *p* = 0.049). Moderate correlations were found between mediolateral center of mass velocity LDE and normalized maximum peak torque (*ρ* = -0.549). This study showed that patients with COPD demonstrate alterations in **s**tride length fluctuations even when adjusted for walking speed, highlighting the potential of nonlinear measures to detect alterations in gait function in patients with COPD. Association with clinical outcomes were moderate to weak, indicating that these clinical test are less discriminative for gait alterations.

## Introduction

Patients with chronic obstructive pulmonary disease (COPD) demonstrate neuromuscular changes [1–4] and gait alterations [5–12]. However, limited studies focus on measures that assess the natural variations that occur from stride-to-stride [10, 12], even less on gait stability [13]. Both measures seem promising for identifying gait alterations within diseases, as has been observed in patients with Parkinson’s Disease [14] and peripheral arterial disease [15]. Moreover, changes in these gait measures may reflect a loss of flexible adaptations of the locomotor system and could be associated with serious accidents like falls. As patients with COPD are at increased fall risk [3], these gait measures seem clinically relevant.

Measures of variability from stride-to-stride have been implicated as indicators of fall risk and future falls [16–18]. Various measures are available to assess stride-to-stride fluctuations. Stride-to-stride fluctuations can be quantified by the standard deviation and coefficient of variation (CoV). Previous study by Yentes et al. [12] reported increased variability in patients with COPD. However, these measures ignore the underlying patterns of stride-to-stride fluctuations over time, which are associated with the acuity of the underlying control [19]. Patterns over many gait cycles can be assessed using sample entropy. Sample entropy quantifies predictability of a time-series [20] and seems reliable for short data sets. An increase in predictability could be association with a loss of flexible adaptations, reflecting a reasonable explanation for the increased occurrence of falls in patients with COPD. Yentes et al. [12] reported reduced sample entropy values in gait in patients with COPD. Another aspect that may be of great importance for gait is gait stability, which can be assessed using the Local Divergence Exponent (LDE, sometimes referred to as maximum Lyapunov exponent). LDE serves as an early indicator for the risk of falling [21] and can differentiate healthy from unhealthy gait patterns in patients with peripheral arterial disease [15]. LDE therefore seems promising for assessing gait stability in patients with COPD as well.

The 6-minute walk test (6MWT) is an important measure of functional exercise capacity and prognosis, and is used as an outcome measure of treatments in patients with COPD [22]. Though the 6-minute walk distance is the primary outcome, in-depth gait assessment could be of added value for clinical purposes to identify gait impairments and risk for falls, and provide targets for gait training. The 6MWT was previously shown to be valid on assessing the walk distance on a self-paced treadmill [23], a feedback controlled treadmill that adapts treadmill speed to its user allowing self-paced walking. Therefore, the aim of this study is to assess stride-to-stride fluctuations and gait stability in patients with COPD and healthy elderly older adults during a self-paced, treadmill 6MWT. It was hypothesized that patients with COPD will show an increased CoV as a measure for the amount of variability, a reduced sample entropy as a measure for predictability of patterns of fluctuations, and an increased LDE (reduced stability) as a measure for stability, as compared to healthy older adults. As COPD also affects function and mobility, it was examined if gait parameters are correlated with functional mobility using the Timed Up and Go (TUG) test and quadriceps muscle strength in patients with COPD.

## Material and methods

### Participants

In this cross-sectional study, a convenience sample of eighty patients with COPD were recruited at a pulmonary rehabilitation center. As controls, thirty-nine healthy older adults were recruited. COPD was determined by spirometry using a post-bronchodilator FEV_1_/Forced vital capacity (FVC) cut-off ratio <0.7. Spirometry (MasterScreen PFT/Body) was conducted prior to the 6MWT. Participants with orthopedic ailments and/or neuromuscular diseases affecting their walking were excluded. Patients requiring walking aids or supplemental oxygen were excluded. Healthy older adults with co-morbidities affecting the pulmonary and/or cardiovascular system were excluded. This study complied with the Declaration of Helsinki and was approved by the Medical Research Ethics Committees United (MEC-U, M13-1374) in the Netherlands. Written consent was obtained from all participants prior to participation.

### Experimental setup

Participants walked on a split-belt instrumented treadmill placed in a virtual reality environment (Gait Real-time Analysis Interactive Lab, GRAIL). This virtual reality environment consisted of a virtual hallway to provide a similar setting as an overground hallway setting [23]. Self-paced treadmill walking was enabled, i.e. the treadmill speed is feedback controlled by the participant, in which the treadmill adjusts its speed to the participant. Previous study by Theunissen et al. [24] reported similar energetics and biomechanics across different speeds of self-paced and fixed speed treadmill walking. The virtual hallway environment was synchronized with the treadmill speed. Reflective markers were placed on anatomical landmarks of each subject according to the Human Body Model (HBM1) of the lower limb [25]. Three-dimensional marker trajectories were recorded using a ten camera Vicon motion capture system (100 Hz). Treadmill force plate data were sampled at 1000 Hz in synchronization with the motion capture system.

Participants performed one familiarization session with a minimum of 3 minutes of walking. Due to the learning effect when repeating tests are performed, two 6MWTs were performed as recommended for field walking tests in chronic respiratory disease [22]. Patients performed the 6MWTs between pre-pulmonary rehabilitation assessment and the first week of pulmonary rehabilitation with a minimum of 45 minutes of rest between exertional tests. Healthy older adults performed the 6MWTs on one day with a minimum of 45 minutes of rest in between the tests [9]. Perceived dyspnea and fatigue (Borg scale), heart rate and pulse oxygen saturation levels were assessed before and after each 6MWT. The instructions for the 6MWT were provided according to the European Respiratory Society/American Thoracic Society guideline [22]. Functional mobility was assessed using the Timed Up & To (TUG) test [26] in patients with COPD. It uses the time that a person takes to rise from a chair, walk three meters, turn around 180 degrees, walk back to the chair, and sit down while turning 180 degrees. The TUG test time ≥12 seconds is associated with falls [27–29] and balance impairment prediction in COPD [30]. Quadriceps muscle strength (peak isokinetic strength) was measured with a Biodex System 3. Patients performed 30 volitional maximal knee extensions at an angular velocity of 90°/sec, while seated upright [31]. The maximum peak torque (Nm) was recorded and normalized maximum peak torque (Nm/kg) was calculated.

### Data processing

The 6MWT with the longest walk distance for each subject and without any stops was used for analysis. The first minute of the data (to minimize start-up effects) and the last 15 seconds (to minimize effects of deceleration of the treadmill) were excluded. Spline interpolation was used to reconstruct all gaps in MATLAB version R2018a (MathWorks Inc., Natick USA). Low pass second order Butterworth filter with a cut-off frequency at 12 Hz was used. Marker and force plate data were processed in custom MATLAB software [9]. Gait events were calculated using a treadmill velocity based method combined with the force plate data, as previously described [9]. Stride time (time from one heel contact to the next ipsilateral heel contact), stride length (distance between the toe marker and the ipsilateral toe marker at each heel contact in the anteroposterior direction, corrected for treadmill speed) and step width (distance between the toe marker in mediolateral direction between both feet at heel strike) were computed. The CoM velocity were derived from the position of the four pelvis markers with respect to time in three directions, e.g. mediolateral, anteroposterior, and vertical. The start and ending were trimmed from the time series. All participants walked a minimum of 444 steps. To include all participants in our analyses and as time series length affects the outcome parameters, time series were cut to the first 444 steps [20, 21]. The CoV quantifies the variability in time series. The CoV was calculated as the standard deviation divided by the mean times 100. The CoV was calculated for stride time, stride length and step width.

Sample entropy quantifies predictability and describes the probability that patterns in a time series are repeated. The method to compute this has been described previously [20]. A perfectly repeatable time series would reflect a sample entropy value of approximately 0, while a completely random time series would reflect a sample entropy value tending towards infinity. It has been suggested that there is a healthy range of entropy that relates to flexibility of movement [32]. Sample entropy was calculated using the *m* parameter, the number of data points that are to be compared; the *r* parameter, the similarity criterion; and the *N* parameter, the length of the entire data set. The relative consistency of the group averages was examined for several combinations of input parameters *r* and *m* [20]. The *r* was chosen as 0.2 times standard deviation of the time series and *m* was chosen as 2 for this study. In contrast to stride length and step width time series, stride time sample entropy was not included for statistical analysis due to the binning effect [33].

Local dynamic stability was assessed by calculating the LDE, e.g. the average logarithmic rate of divergence of adjacent trajectories in state space (see [21]). The LDE has demonstrated theoretical and predictive validity to estimate the probability of falling [34–36]. A lower LDE reflects a more stable system. To reduce problems due to the non-stationarity of the CoM position, velocity data were used instead of position data for the calculation of local dynamic stability. First, the CoM velocity data were normalized to on average 100 samples in length per stride (i.e. 222 strides of data were time-normalized to 22200 samples). Next, the CoM velocity was reconstructed into a multidimensional space using an embedding dimension of 6 for the mediolateral, 7 for the anteroposterior and 6 for the vertical direction. A time delay of 10 samples was chosen as all the time series had a similar main frequency (i.e. the stride frequency, which equaled 1 due to time-normalization). The log of the distances between initially nearest neighbors in state-space were calculated as a function of time and averaged to obtain the average logarithmic rate of divergence. The slope of the divergence curve provided an estimate of the LDE that quantifies the divergence of the CoM velocity trajectories over time [21].

### Statistical analysis

For demographic and clinical characteristics, either an independent t-test or a Mann-Whitney U test were conducted to determine differences between the groups. Group comparisons were performed using an analysis of covariance (ANCOVA) with each gait characteristic as dependent variable (logarithmic transformed), Group as independent variable, and Speed as a covariate to correct for gait speed. Spearman rank correlations were calculated between all gait parameters (mean, CoV, sample entropy, and LDE for stride time, stride length, step width, and CoM velocity), TUG test and normalized maximum peak torque for patients with COPD. A significance level of 0.05 was used. All statistical analyses were performed using SPSS 22.0 software.

## Results

Participants characteristics are presented in Table 1. Walk distance and walking speed was lower in patients with COPD as compared to healthy older adults (Table 2). Pre and post treadmill-based 6MWT pulse oxygen saturation, dyspnea and fatigue levels differed between the groups, except for post heart rate (Table 2). When corrected for Speed, patients had longer stride lengths as compared to healthy older adults (F(1,116) = 4.672, *p* = 0.033; Table 3). Stride length CoV was increased in patients with COPD as compared to healthy older adults after speed correction (F(1,116) = 5.658, *p* = 0.019, Table 3). Patterns within stride length fluctuations were more predictable in patients with COPD as compared to healthy older adults (F(1,116) = 3.959, *p* = 0.049; Table 3. Group differences were not found for LDE (Table 3).

**Table 1:** Demographics of participants.

Characteristics	COPD (*n* = 80)	Healthy (*n* = 39)	*p*
**Age (years)**	62.3 (7.2)	62.1 (6.5)	0.878
**Sex**	48 male/32 female	25 male/14 female	
**Weight (kg)**	75.9 (16.9)	79.1 (12.8)	0.301^a^
**Height (m)**	1.70 (0.09)	1.72 (0.08)	0.159^a^
**Body mass index (kg/m** ^**2**^ **)**	26.3 (5.1)	26.8 (3.1)	0.499
**FEV**_**1**_ **/FVC**	0.41 (0.11)	0.77 (0.05)	< 0.001^a^
**FEV**_**1**_ **(% predicted)**	55.8 (19.4)	119.4 (17.0)	< 0.001
**GOLD I (mild COPD)**	10		
**GOLD II (moderate COPD)**	35		
**GOLD III (severe COPD)**	29		
**GOLD IV (very severe COPD)**	6		
**Pack years**	41.2 (19.2)	8.3 (12.1)	< 0.001^a^
**Never smoker**	1	15	
**Former smoker**	73	23	
**Current smoker**	6	1	
**TUG test (s)**	9.3 (1.3)	7.8 (1.1)	< 0.001
**Maximum peak torque (Nm)**	108.0 (35.6)	125.2 (30.2)	0.013

Data are presented as mean (standard deviation) or number.

^a^: nonparametric test. Abbreviations: GOLD, Global Initiative for Chronic Obstructive Disease.

**Table 2:** The 6-minute walk test outcomes.

Characteristics	COPD (*n* = 80)	Healthy (*n* = 39)	*p*
**6-minute walk distance (m)**	496.7 (79.3)	691.1 (64.4)	< 0.001
**Walking speed (m/s)**	1.4 (0.2)	1.9 (0.2)	< 0.001
**Pre pulse oxygen saturation level (%)**	95.2 (1.5)	97.0 (1.0)	< 0.001^a^
**Post pulse oxygen saturation level (%)**	92.3 (4.6)	97.1 (1.4)	< 0.001^a^
**Pre heart rate (bpm)**	82.1 (13.8)	66.3 (11.9)	< 0.001
**Post heart rate (bpm)**	103.0 (18.1)	98.8 (22.5)	0.309
**Pre dyspnea level**	1.2 (1.2)	0.2 (0.3)	< 0.001^a^
**Post dyspnea level**	4.7 (2.3)	1.1 (1.0)	< 0.001^a^
**Pre fatigue level**	1.4 (1.4)	0.2 (0.4)	< 0.001^a^
**Post fatigue level**	4.5 (2.3)	1.3 (1.1)	< 0.001^a^

Data presented as mean (standard deviation).

^a^: nonparametric test. Abbreviations: bpm, beats per minute.

**Table 3:** Gait parameters of patients with COPD and healthy older adults.

Characteristic	COPD	Healthy	Group	Speed
	(*n* = 80)	(*n* = 39)	F, *p*	F, *p*
**Mean stride time (s)**	1.02 (0.09)	0.89 (0.05)	0.618, 0.434	74.779, < 0.001
**Mean stride length (m)**	1.43 (0.18)	1.73 (0.14)	4.672, 0.033	234.592, < 0.001
**Mean step width (m)**	0.18 (0.04)	0.17 (0.05)	1.513, 0.221	0.080, 0.778
**Stride time CoV (%)**	1.96 (0.77)	1.64 (0.32)	0.273, 0.602	7.020, 0.009
**Stride length CoV (%)**	3.87 (1.78)	2.04 (0.54)	5.658, 0.019	21.396, < 0.001
**Step width CoV (%)**	13.97 (5.35)	17.51 (8.32)	0.028, 0.868	6.346, 0.013
**Stride length sample entropy**	1.12 (0.18)	1.33 (0.10)	3.959, 0.049	7.353, 0.008
**Step width sample entropy**	1.43 (0.04)	1.44 (0.04)	0.202, 0.654	0.984, 0.323
**Mediolateral CoM velocity LDE**	2.87 (0.21)	2.81 (0.23)	1.003, 0.318	6.647, 0.011
**Anteroposterior CoM velocity LDE**	2.84 (0.17)	2.97 (0.24)	0.006, 0.939	6.844, 0.010
**Vertical CoM velocity LDE**	2.80 (0.20)	2.70 (0.23)	0.035, 0.851	2.976, 0.087

Data presented as mean (standard deviation). All values were logarithmic transformed for analysis. Abbreviations: CoM, center of mass; LDE, local divergence exponent.

Within the patient group, stride time was positively correlated with the TUG test (*ρ* = 0.434) and stride length was negatively correlated with the TUG test (*ρ* = -0.506; Table 4). Stride length and step width were both positively correlated with normalized maximum peak torque (*ρ* = 0.477 and *ρ* = 0.249, respectively; Table 4). Weak positive correlations were found between stride time CoV (*ρ* = 0.359), stride length CoV (*ρ* = 0.385), and the TUG test. Step width CoV was negatively correlated with the TUG test (*ρ* = -0.247, *p* < 0.05; Table 4). Stride time CoV was negatively correlated with normalized maximum peak torque (*ρ* = -0.356, *ρ* = -0.368, respectively; Table 4). Sample entropy values of stride length and step width did not correlate with the TUG test or isokinetic peak torque. Anteroposterior CoM velocity LDE and mediolateral CoM velocity LDE had weak to moderate correlations with normalized maximum peak torque (*ρ* = -0.325, *ρ* = -0.549, respectively, Table 4).

**Table 4:** Correlation coefficients between gait parameters and clinical functional outcomes in patients with COPD.

Characteristic	TUG test (s)	Normalized maximum peak torque (Nm/kg)
**Mean stride time (s)**	0.434^b^	-0.066
**Mean stride length (m)**	- 0.506^b^	0.477^b^
**Mean step width (m)**	0.126	0.249^a^
**Stride time CoV (%)**	0.359^b^	-0.368^b^
**Stride length CoV (%)**	0.385^b^	-0.181
**Step width CoV (%)**	- 0.247^a^	-0.161
**Stride length sample entropy**	- 0.154	0.022
**Step width sample entropy**	- 0.086	-0.046
**Mediolateral CoM velocity LDE**	0.152	-0.549^b^
**Anteroposterior CoM velocity LDE**	0.187	-0.325^b^
**Vertical CoM velocity LDE**	- 0.096	0.006

^a^: correlation is significant at the 0.05 level.

^b^: correlation is significant at the 0.01 level.

## Discussion

This study demonstrated that patients with COPD achieved shorter walk distances (i.e. slower walking speed) than healthy older adults, with increased amounts of stride length variability and predictability of stride length patterns. Though only moderate correlations were found between stride time and functional mobility, stride length and functional mobility, and mediolateral CoM velocity LDE and quadriceps muscle strength, treadmill-based gait parameters could possibly be associated with clinical outcomes and could potentially be relevant for clinical practice. These findings support the view that patients with COPD have an altered gait function in comparison with healthy older adults. Local dynamic stability of the CoM velocity did not seem to be affected in patients with COPD when corrected for speed.

Increases in the amount of variability have been associated with impaired gait function. Moreover, variability in gait parameters is associated with increased fall risk in the aging population [17] and in pathological groups [37–39]. Maki et al. [17] observed that increased variability in stride length, speed, and double support was associated independently with falling. Hausdorff et al. [37] reported increased variability in stride time, swing time, and double support time in patients with either Parkinson’s disease or Huntington’s disease compared to healthy participants. Socie et al. [38] demonstrated increased variability in step time and step length in persons with Multiple Sclerosis compared to healthy participants. Increased stride length variability in individuals with Alzheimer disease has been reported [39] and increased fluctuations in step time in patients with COPD was found [12]. These increases in variability may likely reflect a diminished capacity of the locomotor system to adapt to and regulate dynamic conditions [40]. This may result in an inability to compensate for instability, thus predisposing an individual to falls. Our results are in line with these findings and this framework could potentially explain gait alterations in COPD and the increased fall risk in patients with COPD.

Increased predictability (i.e. rigidity) of the locomotor system may be a sign of poor health [15, 40, 41]. Our patients displayed more predictable patterns of stride length fluctuations as compared to healthy older adults. This increase in predictability may provide a possible explanation for the increased occurrence of falls in COPD. In addition, our finding aligns with reduced entropy values in other physiological signals in patients with COPD as compared to healthy individuals, including airflow and heart rate patterns [42].

Patients with COPD are characterized by alterations in gait [5, 6, 8, 9, 11, 12, 43] and diminished functioning [23]. Several factors may underlie the alterations in gait [44]. Skeletal muscle dysfunction, including muscle weakness and impaired muscle metabolism [44], and fatigue [45] could be associated with alterations in stride-to-stride fluctuations in general, however more profoundly in patients with COPD. This study demonstrated that stride length and stability in mediolateral direction are associated with functional mobility and quadriceps muscle function in patients with COPD. Consequently, the altered stride-to-stride fluctuations might be partially reversible following exercise-based pulmonary rehabilitation, which has shown to increase lower-limb muscle strength [46] and functional exercise capacity [22]. A study by Liu et al. [13] demonstrated that a comprehensive pulmonary rehabilitation program did improve mean stride time in patients with COPD, however, stride-to-stride fluctuations (e.g. variability and predictability) of stride time, stride length, and step width did not change. The rehabilitation program used in that study may not have been specific enough to address changes in stride-to-stride fluctuations. Consequently, it is of interest to further identify the relationship between stride-to-stride fluctuations and clinical functional outcome parameters, to adjust the rehabilitation program for patients experiencing walking difficulties.

Falls in the aging population have been associated with stride-to-stride fluctuation measures, such as variability in time series (e.g. CoV) [17, 18], and stability measures, such as LDE [21]. Increased variability of stride lengths may increase the risk of falling during walking, for example, due to errors in foot placement and/or center of mass displacement [17]. Changes in the patterns within stride-to-stride fluctuations may reflect a loss of flexible adaptations within the locomotor system, indicating gait impairments. Within the aging population, patients with COPD have a higher risk for falls [3]. Alterations in stride-to-stride fluctuations, and patterns of these fluctuations, are therefore of interest to identify the relationship with fall risk in this specific patient population. Once this relationship is established, the CoV and sample entropy might be used as indicators for gait function and as targets for specific training programs.

In the current study, however, patients with COPD did not demonstrate reduced local dynamic stability compared to healthy older adults when corrected for speed. This is in contrast with reduced local dynamic stability as found in the aging population [36]. A possible explanation could be that our patients were relatively in good condition and could have exhibited less gait and balance impairments, as they were able to perform the self-paced treadmill 6MWT without any stops or falls. Future studies are therefore recommended exploring stability measures in patients with COPD or exposing patients to different walking conditions, as this measure has been proposed as an indicator for fall risk [21, 36].

Though LDE did not differ between the groups, significant correlations were found between LDE and normalized quadriceps strength. These results imply that patients with COPD with higher maximum peak torque values had lower LDE-values in mediolateral and anteroposterior direction, suggesting a higher gait stability during the 6MWT. As weakness of quadriceps muscles in the aging population is associated with falls [47], the role of this muscle group should be considered for maintaining gait stability in patients with COPD.

The TUG test was significantly correlated with mean stride time and stride length, indicating that patients with increased stride time and shorter stride lengths achieve worse TUG test results. CoV was significantly correlated with TUG test, indicating that patients with poor results on the TUG test might demonstrate gait impairments. However, only weak correlations were found. A possible explanation could be that patients were in relatively good condition. None of the patients stopped walking during the 6MWTs and none used walking aids. The results of the TUG test support this assertion, as patients did not demonstrate abnormal results on the TUG test of 11.2 seconds or above [28].

### Study limitations

Several limitations of this study should be mentioned. The first limitation is that patients in Global Initiative for Chronic Obstructive Disease (GOLD) 1 (mild COPD) and GOLD 4 (very severe COPD) categories [48] were less represented in this study. Patients classified into GOLD stage 4 more frequently used oxygen and/or were not able to perform the self-paced, treadmill 6MWT without any stops or using the handrails. In addition, walking with handrails would have altered gait pattern of participants, e.g. stabilizing the center of mass during the test. Patients classified into GOLD stage 1 are generally less likely to be referred for pulmonary rehabilitation, due to a lower burden of disease. The patients in this study do not reflect the heterogenic COPD patient population within all GOLD stages. Therefore, some caution is needed to interpret our results. The second limitation is that the effect of optic flow provided by the virtual reality environment was not examined in the specific patient or age group. This could have presented a challenge or helped those due to the increase in visual information during walking. Consequently, our results should be interpreted with some caution. However, a previous study assessed self-paced treadmill walking and virtual reality in healthy young adults. The effects of virtual reality on gait were too small to be relevant and participants reported walking with virtual reality as more similar to overground walking [49]. The third limitation is the adaptation to self-paced treadmill walking. Participants walked a minimum of 3 minutes to familiarize with self-paced treadmill walking. Due to the symptoms patients with COPD experience and their limited exercise capacity, they may not have been completely accustomed to adjust their walking speed. Though self-paced treadmill walking has been studied in healthy participants [24, 50], it has not been tested in this patient or age group. In addition, all participants performed the 6-minute walk test twice and the second test was most often used for analysis.

## Conclusions

Patients with COPD demonstrate more variable stride lengths, while stride length patterns were more predictable as compared to healthy older adults during the self-paced treadmill 6MWT. Current findings highlight the potential of stride-to-stride fluctuations and gait stability measures to detect alterations in gait function in patients with COPD. Association with clinical outcomes were moderate to weak, indicating that these clinical test are less indicative of gait alterations. Gait analysis may provide targets for the development and evaluation of training program interventions aimed at reducing falls, improving mobility or reducing fatigue.

## Abbreviations

6MWT: 6-minute walk test
CoM: center of mass
COPD: chronic obstructive pulmonary disease
CoV: coefficient of variation
FEV_1_: Forced Expiratory Volume in the first second
FVC: Forced Vital Capacity
GRAIL: Gait Real-time Analysis Interactive Lab
LDE: Local Divergence Exponent
TUG test: Timed Up and Go Test
